# Two co-inherited SNPs of the telomerase reverse transcriptase (TERT) gene are associated with Iraqi patients with lung cancer

**DOI:** 10.5937/jomb0-41553

**Published:** 2023-10-27

**Authors:** Zahraa K. Lawi, Ahmed H. Alkhammas, Malek Elerouri, Amara Ibtissem Ben, Mohammed Baqur S. Al-Shuhaib

**Affiliations:** 1 University of Kufa, College of Science, Department of Biology, Najaf, Iraq; 2 University of Sfax, Faculty of Science, Department of Biology, Sfax, Tunisia; 3 Al-Qasim Green University, College of Agriculture, Department of Animal Production, Al-Qasim, Iraq; 4 University of Sfax, National Engineering School in Sfax, Department of Enzyme Engineering and Microbiology, Sfax, Tunisia; 5 University of Sfax, Higher Institute of Biotechnology, Laboratory of Medicinal and Environment Chemistry, Sfax, Tunisia; 6 University of Warith Al-Anbiyaa, College of Medicine, Karbala, Iraq

**Keywords:** TERT, polymorphism, NSCLC, rs2736098, rs10069690, TERT, polimorfizam, NSCLC, rs2736098, rs10069690

## Abstract

**Background:**

The telomerase reverse transcriptase (TERT) gene is essential polymorphic loci linked to most malignant tumors. This study assessed the association between the TERT gene and non-small cell lung carcinoma (NSCLC) in Iraq.

**Methods:**

Genomic DNA samples were extracted from a total of 200 samples of blood. Four specific PCR fragments were designed to amplify four high-frequency rs2735940, rs2736098, rs2736100, and rs10069690 SNPs within the TERT gene. Single-strand conformation polymorphism (SSCP) followed by sequencing reactions were used for genotyping and validating the amplified fragments.

## Abstract (sr)

**Uvod:**

GEN za reverznu transkriptazu telomeraze (TERT) je esencijalni polimorfni lokus povezan sa većinom malignih tumora. Ova studija je procenila vezu između TERT gena i karcinoma pluća ne-malih ćelija (NSCLC) u Iraku.

**Metode:**

Uzorci genomske DNK su ekstrahovani iz ukupno 200 uzoraka krvi. Četiri specifična PCR fragmenta su dizajnirana da amplifikuju četiri visokofrekventna rs2735940, rs2736098, rs2736100 i rs10069690 SNP u okviru TERT gena. Za genotipizaciju i validaciju amplifikovanih fragmenata korišćen je jednolančani konformacioni polimorfizam (SSCP) praćen reakcijama sekvenciranja.

**Rezultati:**

Pacijenti sa genotipom rs2735940: A/G su bili u značajno većem riziku od razvoja NSCLC (P=0,0299; OD 2,3158; Cl95% 1,0853 do 4,9414). Pojedinci sa genotipom rs2736098: C/T su takođe značajno povezani sa povećanom verovatnoćom razvoja NSCLC (P=0,0363; OD 2,1583; Cl95% 1,0503 do 4,4351). Analiza neravnoteže veze je pokazala da su oba SNP-a pokazala veoma visok nivo konasleđa pacijenata. LD dijagram je pokazao da je alel T od rs2736098 sarađivao sa alelom G od rs2735940 da generiše TG haplotip kod pacijenata. Prema našim nalazima, utvrđeno je da su i TERTrs2735940: A/G i TERT-rs2736098: C/T SNP u značajnoj vezi sa povećanim rizikom od NSCLC. Oba SNP-a su pokazala najveće vrednosti ko-nasleđivanja kod pacijenata. Ovo ko-nasleđivanje je uglavnom predstavljeno alelima rs2735940: A i rs2736098: C. I patogeni T i G aleli su generisali TG haplotip koji je dostupan samo u uzorcima pacijenata.

**Zaključak:**

Ova studija predlaže korišćenje haplotipa TG kao biomarkera koji obećava u smislu rane dijagnoze NSCLC. Potrebna je dalja validacija ovih nalaza opsežnim istraživanjem na većoj populaciji.

## Introduction

Lung cancer is one of the most severe public health challenges and a major contributor to cancer-related morbidities and fatalities worldwide. Lung cancer cases are being reported more frequently in the Middle East regions [1]. Recent years have shown a steady increase in the clinical risk factors for lung cancer and its associated consequences [2]. It has been demonstrated that traditional methods of diagnosing lung cancers are less effective due to several issues related to higher mortality rates reported in developing malignant lung tumors in their advanced stages [3]. Non-small cell lung cancer (NSCLC) is one of the most critical types of respiratory tract tumours, making up 80% of lung carcinogenesis worldwide. Unfortunately, it has been reported that surgical treatment may not be possible if NSCLC develops in severe stages III and IV [4]. As a result, NSCLC is substantially more challenging to detect and cure early than other types of cancer [5]. This consequence is linked to people with lung cancer, typically detected in advanced stages, having a poor prognosis [6]. Given the sharp decline in lung cancer survival rates from early to advanced stages, more focus should be given to this issue regarding its early diagnosis [7]. Because of this, it is critical to detect this type of cancer early by integrating molecular research with traditional clinical evaluations [8] [9]. Recently, the onset and progression of lung cancer have been linked to various extents by a wide range of genetic variants [10] [11]. The telomerase reverse transcriptase (*TERT*) gene is one of the essential polymorphic loci linked to various human cancers [12]. The *TERT* gene has 16 exons and is positioned in chromosome 5 (5p15.33). The *TERT* gene encodes the catalytic component of telomerase, a ribonucleoprotein that plays a pivotal role in cancer initiation via telomere-dependent or telomere-independent pathways [13]. The methods for maintaining telomeres entail intricate cellular adjustments brought on by TERT, such as TERT structural variants [14], *TERT* gene amplifications [15], *TERT* epigenetic changes [16], and alternative lengthening of telomere [17], and *TERT* promoter mutations [18]. Any alteration of these mechanisms is possibly associated with numerous types of carcinogenesis at a single-cell level [14]. Telomere length is influenced by TERT-based telomerase activity, which can also act as an informative biomarker in the early diagnosis and prognosis of many malignancies [19]. TERT aids telomerase in replenishing telomere sequences by extending the extreme ends of telomere strands to enable other polymerases to synthesize the complementary strand [20]. It has been established that elevated TERT expression leads to telomerase activity restoration [21], implying that the transcriptional control of TERT plays a remarkable role in carcinogenesis [22]. Due to its significance in numerous critical actions throughout the cells, *TERT* polymorphism may be connected to the early onset and progression of malignancies. It has been shown that the *TERT* gene is linked to a diverse spectrum of malignancies, such as pancreatic cancer [23], prostate cancer [24], gastric cancer [25], thyroid cancer [26], esophageal cancer [27], and bladder cancer [28], melanoma [29]. However, controversial data have been found on the possible association of the *TERT* gene with lung cancers [4]. This is due to the poor association with squamous cell carcinoma and NSCLC [30] [31]. But later on, many sources of data have shown that there is a strong connection between *TERT* variants and the risk of NSCLC [32] [33] [34]. In Iraq, the prevalence of NSCLC has steadily increased. This is due to exposure of Iraqi people the several disastrous wars and the deterioration of health care infrastructures [35]. However, the significance of several *TERT* SNPs in the development of NSCLC have not been identified in this population. Taking these data into consideration, this research aim was to investigate the possible association between four *TERT* high-frequency SNPs rs2735940, rs2736098, rs2736100, and rs10069690 with increased risk of NSCLC in cancer subjects in Iraq.

## Materials and methods

### Controls and Subjects

The experiments performed in the study were conducted following the Helsinki Declaration for experiments involving people, and the biochemical research involving human subjects was approved by the Institutional Review Board (IRB) in the University of Kufa (IECIH/UOK 088/2020; CAAE 08802212). Signed written informed consent was obtained from all participants before being involved in the study. In this case-control study, a total of 200 individuals, of which 100 patients with NSCLC and 100 controls, were included. The details of the collected samples were described in [36]. Briefly, the samples of patients were collected from the Middle-Euphrates Cancer Center (located in Najaf governorate) and Merjan Cancer Center (located in Babil governorate). The involved patients ranged in age from 24 to 80 (average age: 52.2). Each had previously received a diagnosis of NSCLC from trained personnel to diagnose malignancies at the facilities mentioned above in the study area. In this investigation, 100 healthy volunteers with no prior history of lung cancer and ages ranging from 18 to 78 made up the control population (mean age: 44.4). The samples that were screened all belonged to citizens of Iraq, and they were all examined from February to December of 2021.

### Genomic DNA extraction

Genomic DNA samples were extracted from the peripheral blood samples using a Blood/Cell DNA Mini Kit (Cat. No. GB100, Geneaid Co., Taipei, Taiwan). A Nanodrop spectrophotometric technique was used to confirm the purity of the extracted genomic DNA (Biodrop Co., UK). The isolated genomic DNA's integrity was assessed by agarose gel electrophoresis following the recommended standard instructions [37].

### PCR

**Figure 1:**
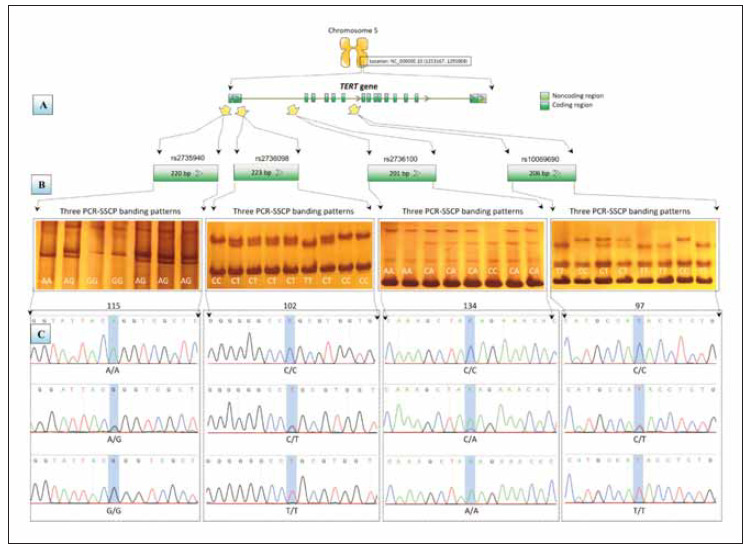
A schematic diagram of TERT genotyping using the PCR-SSCP-sequencing method. A) – PCR designing of four PCR-specific primers for the amplification of 220 bp, 223 bp, 201 bp, and 206 bp to flank rs2735940, rs2736098, rs2736100, and rs10069690, respectively. B – PCR-SSCP genotyping, in which all targeted SNPs showed three polymorphic patterns of nucleic acid variations. C – Sequencing reactions of the targeted loci as positioned in the amplified PCR fragments

Four sets of PCR-specific primers were designed using NCBI primer BLAST tool to amplify four distinct PCR fragments within the *TERT* gene [38]. In PCR designing, four high-frequency SNPs (rs2735940, rs2736098, rs2736100, and rs10069690) were targeted within total lengths of 220 bp, 223 bp, 201 bp, and 206 bp, respectively (Figure 1a). The specified features of PCR products that are required to provide the best resolutions in PCR-SSCP methods were met by the PCR amplicons when they were prepared with these lengths [39]. The details of the PCR oligonucleotide sequences are displayed in Table 1. The experiments of PCR were conducted by a lyophilized PCR AccuPower PreMix (Cat# K-2012, Bioneer Co., Korea), with a final volume of 20 μL for each amplified fragment. After performing PCR experiments, it was confirmed that PCR products were in the expected sizes by electrophoresis on agarose gels.

**Table 1:** The specific PCR primers designed for the amplifiation of the TERT gene. PCR primers were designed based on the GenBank acc. no. NC_000005.10

Targeted<br> SNP	SNP locus in<br> the amplicon	Primer’s<br> direction	Primer’s<br> code	Sequence (5-3)	Length<br> (bp)	PCR<br> conditions
rs2735940	115	Forward	TERT-1F	CTGCCGGAGGAAATTGCTTT	220	60.1 °C
		Reverse	TERT-1R	CAAATTTTCCAAACCGCCCCT		
rs2736098	102	Forward	TERT-2F	TCCTTGTCGCCTGAGGAGTA	223	61.0 °C
		Reverse	TERT-2R	CGAGTGACCGTGGTTTCTGT		
rs2736100	134	Forward	TERT-3F	AAAGCTGGGGTTTACCAGCAA	201	59.9 °C
		Reverse	TERT-3R	TCTCAGGCATCTTGACACCC		
rs10069690	97	Forward	TERT-4F	CCAGACCCGGGACCTAGAA	206	60.1 °C
		Reverse	TERT-4R	CTGAGGATCCTGGACCTTGC		

### Genotyping analysis

The genotyping experiments were conducted using PCR-single strand conformation polymorphism (SSCP). This sensitive and inexpensive genotyping approach can discriminate between PCR products that differ in only one nitrogen base using denaturation, chilling, and electrophoresis on polyacrylamide gels [40]. Briefly, PCR amplicons were denatured at 94 for 7 to 8 min and then immediately frozen in ice for at least 10 min. Then, PCR products were subsequently loaded in 8% polyacrylamide gels until samples reached the bottom of the gels. Gels were stained with silver nitrate following the recommended procedure [41]. Each detected SSCP banding pattern was subjected to Sanger dideoxy-sequencing laboratories to confirm the obtained electrophoretic genotypes following the recommended instructions (Macrogen Inc., South Korea). The DNA chromatogram of each genotype was visualized using SnapGene Viewer ver. 4.0.4. (Insightful Science, Canada). The alignment of the observed variation with the referring DNA sequences was conducted using BioEdit suit, version 7.1 (DNASTAR; Madison, USA).

### Statistical and functional analysis

Using the MedCalc online server, the odds ratios (ORs) and associated 95% confidence intervals (CIs) were computed to examine the genotype variations for the targeted SNPs between the patients and controls [42]. Gen-Calculator software (www.genecalc.pl) was utilized to evaluate the likelihood that both the cases and the controls would deviate from the Hardy-Weinberg equilibrium (HWE). The linkage disequilibrium (LD) plots of the identified SNPs were constructed by Haploview software ver. 4.2 to assess the likely prevalence of haplotypes' co-inheritance in both controls and patients [43]. The p-values below 0.001 were regarded as significant. After retrieving the positions of investigated SNPs from the reference genomic sequence, the possible connections between their haplotypes were assessed.

## Results

### Genotyping analysis

After reviewing the SNPs database for the *TERT* gene according to their frequency in the dbSNP web server, four SNPs with the highest frequency were selected respectively. These four SNPs are rs2735940, rs2736098, rs2736100, and rs10069690, which were chosen to be screened in four distinct positions of the *TERT* gene. Three different banding patterns were found for each selected SNP, demonstrating that the rs2735940, rs2736098, rs2736100, and rs10069690 SNPs each have three genotypes (Figure 1b). The conducted sequencing experiments verified the three expected genotypes for all investigated high-frequency SNPs. The electropherograms of the four investigated SNPs demonstrated these three genotypes (rs2735940: AA: AG: GG, rs2736098CC: CT: TT, rs2736100: CC: CA: AA, and rs10069690: CC: CT: TT) (Figure 1c).

To determine if the study population was in HWE, the genetic diversity of the identified polymorphic SNPs was performed. According to the chi-square values with Yate's corrections, the polymorphisms of all four detected polymorphic SNPs were confirmed to be compatible with HWE in both control and NSCLC groups at the significance level of 0.05 (Table 2).

**Table 2:** Hardy-Weinberg equilibrium (HWE) for rs2735940, rs2736098, rs2736100, and rs10069690 SNPs of the TERT gene in patients and control groups The P-value with statistical significance is in bold. CL – confidence interval.

SNP	Patients		Controls	
rs2735940 (A115G)	Observed	Expected	Observed	Expected
AA	68	65.61	78	75.31
AG	26	30.78	21	26.38
GG	06	03.61	05	02.31
Chi-square value	2.41167		4.32537	
P-value	0.29944		0.11502	
rs2736098 (C102T)	Observed	Expected	Observed	Expected
CC	66	62.41	82	79.21
CT	26	33.18	14	19.58
TT	08	4.41	04	1.21
Chi-square value	4.6827		8.12163	
P-value	0.0962		0.01723	
Yate’s chi-square value			5.71817	
Yate’s P-value			0.05732	
rs2736100 (C134A)	Observed	Expected	Observed	Expected
CC	54	50	59	55.8
CA	32	40	31	38
AA	12	8	10	6.5
Chi-square value	3.92		3.3894	
P-value	0.14086		0.18365	
rs10069690 (C97T)	Observed	Expected	Observed	Expected
CC	70	67.24	85	82.81
CT	24	29.52	12	16.38
TT	6	3.24	3	0.81
Chi-square value	3.4966		7.15024	
P-value	0.17407		0.02801	
Yate’s chi-square value			4.47961	
Yate’s P-value			0.10648	

### Association analysis

The nucleic acid differences of four investigated SNPs were compared between controls and patients to determine whether there may be an association between the *TERT* gene and NSCLC (Table 3).

**Table 3:** Association of rs2735940, rs2736098, rs2736100, and rs10069690SNPs with the risk of NSCLC The P-values with statistical significance are shown in bold. CL – confidence interval.

SNP	Patients (n = 100)	Controls (n = 100)	Odds ratio (95% Cl)	P-value
rs2735940 (A115G)				
AA	70 (70%)	85 (85%)	Reference value	
AG	24 (24%)	12 (12%)	2.3158	0.0299
GG	06 (06%)	03 (03%)	2.0638	0.3154
A allele	164 (82%)	182 (91%)	Reference value	
G allele	036 (18%)	018 (09%)	2.2195	0.0097
rs2736098 (C102T)				
CC	66 (66%)	82 (82%)	Reference value	
CT	26 (26%)	14 (14%)	2.1583	0.0363
TT	08 (08%)	04 (04%)	2.0870	0.2425
C allele	158 (79%)	178 (89%)	Reference value	
T allele	042 (21%)	022 (11%)	2.1507	0.0072
rs2736100 (C134A)				
CC	54 (54%)	59 (59%)	Reference value	
CA	32 (32%)	31 (31%)	1.0474	0.8790
AA	14 (14%)	10 (10%)	1.4651	0.3861
C allele	140 (70%)	149 (74.5%)	Reference value	
A allele	60 (30%)	051 (25.5%)	1.2521	0.3153
rs10069690 (C97T)				
CC	68 (68%)	78 (78%)	Reference value	
CT	26 (26%)	21 (21%)	1.3218	0.4051
TT	06 (06%)	05 (05%)	1.2128	0.7567
C allele	162 (81%)	177 (88.5%)	Reference value	
T allele	038 (19%)	031 (15.5%)	1.2788	0.3549

For rs2735940 (G/A) SNP, It was observed that the heterozygous AG genotype was significantly more frequent in patients than in controls. Thus, individuals with the heterozygous AG genotype showed a significantly higher risk of developing NSCLC with a *P*-value of 0.0299 (OD 2.3158, Cl_95%_ 1.0853–4.9414). This data indicated that individuals with allele G had a considerably higher risk of developing NSCLC with a *P*-value of 0.0097 (OR 2.2195; Cl_95%_ 1.2134–4.0599). For rs2736098 (C/T) SNP, noticeable differences were identified in the distributions of the heterozygous CT genotype between the patients and controls. Individuals with the CT genotype exhibited a significantly higher risk of developing NSCLC with a *P*-value of 0.0363 (OD 2.1583, Cl_95%_ 1.0503–4.4351). This data also showed that individuals with allele T exhibited a significantly higher risk of developing NSCLC with a *P*-value of 0.0072 (OR 2.1507; Cl_95%_ 1.2303–3.7598). Regarding the other two SNPs under investigation (rs2736100 and rs10069690), no significant associations were identified in their alleles and genotypes distribution between the patients and controls. rs2736100 (CA) exerted no obvious preferences for any genotype or allele to be in patients or controls since no significant association was detected in the distribution of this SNP between both involved groups (*P*-value 0.8790 and 0.3861 for CA and AA genotypes, respectively). The same observation also applied to the distribution of rs10069690 (CT). Though the heterozygous genotype CT showed a higher frequency in patients (26%) than the controls (21%), The *p*-value analysis reflected statistically insignificant differences between the patients and controls for this SNP (*P*-value 0.4051).

### LD analysis

**Figure 2:**
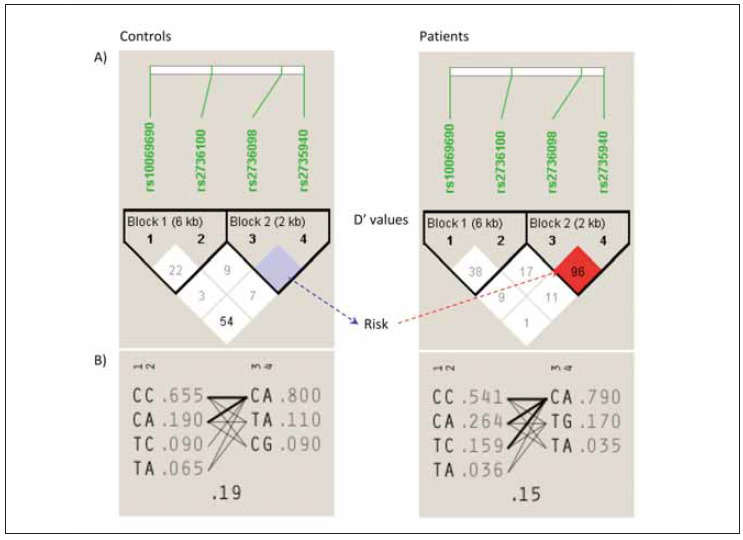
Haplotype block map for four investigated SNPs in the TERT gene. Standard color schemes are used to display LD values. Bright red colour indicates very strong LD (LOD > 2, D’=1), pink colour indicates less strong LD (LOD > 2, D’< 1), and blue colour indicates partial linkage (LOD < 2, D’=1), and white colour indicates complete recombination (LOD < 2, D’< 1)

The observed haplotypes were evaluated, and the values of linkage disequilibrium were determined between the four polymorphic loci in the current population to assess the co-inheritance potential among them. The observed D' (1.0) and LOD (0.96) values showed the presence of partial linkage between two polymorphic SNPs in the controls, namely rs2735940 and rs2736098. Whereas the other values of the other two SNPs showed no evidence of coinheritance (Figure 2a). In the patients' population, LD plot analysis showed that the rs2735940 and rs2736098 were highly co-inherited (D' 0.96, LOD 22.6) (Table 4).

**Table 4:** The computed linkage values four investigated four SNPs in the TERT gene as determined by LD plot analysis

	L1	L2	D’	LOD	r^2	CIlow	CIhi	Dist	T-int
For controls	rs10069690	rs2736100	0.22	0.75	0.026	0.04	0.43	6726	1.01
	rs10069690	rs2736098	0.039	0.03	0.001	-0.01	0.26	14296	–
	rs10069690	rs2735940	0.54	0.23	0.005	0.05	0.88	16696	–
	rs2736100	rs2736098	0.096	0.08	0.003	0.0	0.39	7570	0.4
	rs2736100	rs2735940	0.073	0.06	0.002	0.0	0.36	9970	–
	rs2736098	rs2735940	1.0	0.96	0.012	0.14	0.99	2400	1.25
For patients	rs10069690	rs2736100	0.382	0.5	0.015	0.05	0.68	6726	0.52
	rs10069690	rs2736098	0.095	0.02	0.001	0.01	0.61	14296	–
	rs10069690	rs2735940	0.011	0.0	0.0	-0.01	0.22	16696	–
	rs2736100	rs2736098	0.175	0.57	0.018	0.02	0.36	7570	0.78
	rs2736100	rs2735940	0.112	0.19	0.006	0.0	0.33	9970	–
	rs2736098	rs2735940	0.963	22.6	0.763	0.85	1.0	2400	22.79

The haplotype analysis further details the LD plot among the investigated polymorphic loci. This high value entailed a high genetic collaboration between rs2735940 and rs2736098 in the patients' group. Based on their positions occupied within the genomic sequences of the *TERT* gene, these four SNPs were separated accordingly into two adjacent blocks. The 6 kb distance block-1 was created by combining rs2736100 and rs10069690. Whereas block-2 of 2 kb distance was created between rs2735940 and rs2736098 SNPs. In controls, the highest ratio of haplotypes' distributions between both blocks was represented by CC: CA and CA: CA, with a multi-allelic D of 0.19. Whereas the highest ratio of haplotypes' distributions between both involved blocks were CC: CA, CA: CA, and TC: CA, with a multi-allelic D of 0.15. LD plot showed that the observed CA haplotype represented the highest distribution of 0.8 to 0.79 in controls and patients, respectively (Figure 2b). This haplotype was generated by the collaboration of both rs2735940 (AG) and rs2736098 (CT) SNPs in the block-2 in both populations. Interestingly, it was found that the collaboration between the pathogenic T allele in rs2736098 SNP with the pathogenic G allele in rs2735940 SNP created the TG haplotype. This haplotype is patient-specific and not found in the control population.

## Discussion

One of the main reasons for the high mortality rates associated with lung cancer in many parts of the world is related to its inadequate early detection [44]. Thus, it is critical to develop useful genetic biomarkers for the early diagnosis of lung cancer [45]. The *TERT* gene is one of these intriguing factors that has been shown to have a remarkable connection with the initiation and development of many cancers. Many *TERT* biomarkers have been discovered to be risk factors for NSCLC. In various populations affected by the steady increase in these cases, it is unclear if many certain alleles of the *TERT* gene pose a risk for NSCLC individuals. This study described the connection between TERT polymorphism and NSCLC in the Iraqi population by genotyping four high-frequency SNPs positioned in four regions within the *TERT* gene.

Out of four investigated SNPs, the polymorphisms of rs2736100 and rs10069690 SNPs did not show any significant association with NSCLC. This finding implies no evidence of a connection between these variants and lung cancer in the investigated population. However, our results do not align with other research that indicated the significant association of rs2736100 and rs10069690 SNPs with lung cancer in Chinese and Korean populations [34] [46] [47] [48] [49] [50] [51]. This may be due to the lower number of investigated samples that represent the main limitation of this case-control study. However, genotyping 100 patients with NSCLC is tedious due to many logistic issues related to the strict identification of this type of cancer, and eliminating other types of lung cancers has reduced the sample size.

In contrast to rs2736100 and rs10069690 SNPs, this study found that both rs2735940 and rs2736098 SNPs showed significant associations with the development of NSCLC in the investigated Iraqi subjects. This association may indicate the potential roles of these SNPs in the progression of NSCLC. In agreement with our results, many reports have shown a significant association between the polymorphism of rs2735940 SNP and the development of lung cancers in several populations, including Chinese [52], Koreans [53], Icelanders [54], and African-Americans [55]. In contrast, this SNP is also reported to be associated with a decreased risk of lung cancer in American women [56]. However, the inclusion of one gender in the case-control investigation may change the genotype-phenotype association. In addition to lung cancer, rs2735940 SNP has also been associated with multiple malignancies, such as renal cell carcinoma [57], head and neck cancer [31], and gastric cancer [25]. Furthermore, rs2735940 SNP showed variable distributions in many populations, which refers to variable biological diversity. According to dbSNP, the rs2735940 SNP allele G is deposited with high frequencies in Latin Americans (G=0.54 to 0.63), East Asians (G=0.53), Asians (G=0.52), Africans (G=0.49), and Europeans (G=0.48). Whereas it is deposited in lower frequencies in South Asians (G=0.32). However, the population of the Middle East has the lowest frequencies of the allele G, with only 0.26 in the Qatari population.

Although rs2736098 SNP is less known than rs2735940 SNP, it is also linked to the development of lung cancer [32] [53]. Moreover, it is also linked with the increased risks of other cancers [58], such as nasopharyngeal carcinoma [59], hepatocellular carcinoma [60], cervical carcinoma [61], and breast cancer [62]. The rs2736098 SNP allele T is deposited in dbSNP with lower frequencies than in rs2735940 SNP. This can easily be shown in the percentage of T allele that deposited in 0.45, 0.41, 0.38, 0.27, 0.22-0.19, and 0.11 in South Asians, Asians, East Asians, Europeans, Latin Americans, and Africans, respectively. Noteworthy, the A allele was deposited in the Qatari population at the same frequency in which the G allele of rs2735940 SNP was deposited (0.26). This point may support our findings of the presence of strong collaboration between both polymorphic loci in the studied area of the Middle East.

Both the rs2736098 and rs2735940 SNPs have been deposited in the ClinVar database because of their significance in developing many different forms of tumors [63]. Due to the synonymous effect of rs2735940 SNP, it seems that this variant exhibits its effect by modulating splicing patterns [64] [65], regulating the velocity of mRNA translation [66], or influencing the protein kinetics [67]. Whereas the effect of the 5 -UTR rs2736098 SNP differs based on its different position upstream of the open reading frame. It was reported that 5 -UTR SNPs effects are mainly concerned with altering mRNA translation and decay [68]. Whatever the mechanism through which each SNP impacts the TERT, both rs2736098 and rs2735940 SNPs showed a high collaboration in the progression of NSCLC. This can be seen in the heterozygous genotypes of rs2736098: CT and rs2735940: AG as both showed a remarkable tendency to be co-inherited with each other the study population. Further analysis of haplotype distributions revealed that both SNPs are mainly co-inherited by the CA haplotype. In the patient population, the CA haplotype has a greater contribution with further genetic formulations than that found in the control group. This haplotype was generated by the contribution of the C and A alleles from rs2736098 and rs2735940 SNPs, respectively. Most importantly, the pathogenic T allele of rs2735940 and the pathogenic G allele of rs2736098 SNPs have collaborated to generate the TG haplotype, a distinct haplotype for patients. Due to the absence of TG haplotype in control, it is rather important to figure out its severity in association with NSCLC in the studied population. This sort of cooperation may be attributed to the close positioning of both SNPs in the *TERT* gene sequences. To the best of our knowledge, no previous report has claimed this sort of co-inheritance between rs2736098 and rs2735940 SNPs in the *TERT* gene. However, more pathological connections between *TERT* gene and NSCLC are need to be clarified the get further details on the mechanisms of this con-inheritance.

In conclusion, the genotyping experiments conducted on four variants within the *TERT* gene indicated the presence of a significant association between the polymorphisms of rs2735940 and rs2736098 SNPs and NSCLC in the studied Iraqi subjects. Individuals with rs2735940: AG and rs2736098: CT genotypes exhibited higher risks of NSCLC. This research demonstrated that both AG and CT genotypes collaborated to generate a high level of co-inheritance represented by the haplotype TG. This haplotype is specific for patients and may be associated with the development of NSCLC. Accordingly, the haplotype TG can serve as a promising biomarker for the early diagnosis of NSCLC. However, large-scale screening experiments are suggested to provide another layer of confirmation of the current findings.

## Dodatak

### Funding

This work was part of the Ph.D. thesis of ZKA prepared at the Higher Institute of Biotechnology of Sfax, 3000 Sfax University, Tunisia (IQ200476). The authors declare that no funds, grants, or other support were received during the preparation of this manuscript.

### Data availability statement

The data supporting this study's findings are available on request from the corresponding author. The data are not publicly available for privacy and ethical restrictions, as stipulated by the University of Kufa Institutional Review Board.

### Authorship contribution statement

ZKL developed the work and conducted genotyping experiments. TMA supervised the research. AHA and ME participated in the genotyping experiments. MBSA contributed to the supervision of the work, analyzed the data, and wrote the manuscript. All authors approved the final version of the work.

### Acknowledgments

The authors thank the Middle-Euphrates Cancer Center (MECC), Najaf province, and Merjan Cancer Center (MCC), Babil province, and the postgraduate laboratory unit, College of Medicine, University of Kufa for granting the samples and providing the informative scientific help to accompany the preliminary data preparations.

### Conflict of interest statement

All the authors declare that they have no conflict of interest in this work.

## References

[1] Jazieh A R, Algwaiz G, Errihani H, Elghissassi I, Mula-Hussain L, Bawazir A A, Gaafar R (2019). Lung cancer in the Middle East and North Africa region. J Thorac Oncol.

[2] Hussain A M A, Lafta R K (2021). Cancer trends in Iraq 2000-2016. Oman Med J.

[3] Wong M, Lao X Q, Ho K - F, Goggins W B, Tse S L A (2017). Incidence and mortality of lung cancer: Global trends and association with socioeconomic status. Sci Rep.

[4] Zhao X, Wang S, Wu J, Li X, Wang X, Gao Z, Wu W, Wang H, Wang J, Qian J, Ma K, Li H, Han B, Bai C, Li Q, Liu W, Lu D (2015). Association of TERT polymorphisms with clinical outcome of non-small cell lung cancer patients. PLoS One.

[5] Gao H, Niu Y, Li M, Fang S, Guo L (2017). Identification of DJ-1 as a contributor to multidrug resistance in human small-cell lung cancer using proteomic analysis. Int J Exp Pathol.

[6] Henschke C I, McCauley D I, Yankelevitz D F, Naidich D P, McGuinness G, Miettinen O S, Libby D M, Pasmantier M W, Koizumi J, Altorki N K, Smith J P (1999). Early lung cancer action project: Overall design and findings from baseline screening. Lancet.

[7] Diaz-Lagares A, Mendez-Gonzalez J, Hervas D, Saigi M, Pajares M J, Garcia D, Crujerias A B, Pio R, Montuenga L M, Zulueta J, Nadal E, Rosell A, Esteller M, Sandoval J (2016). A novel epigenetic signature for early diagnosis in lung cancer. Clin Cancer Res.

[8] Hamann H A, Ver Hoeve E S, Carter-Harris L, Studts J L, Ostroff J S (2018). Multilevel opportunities to address lung cancer stigma across the cancer control continuum. J Thorac Oncol.

[9] El-Baz A, Gimel'farb G, Falk R, Abou E M, Rainey S, Heredia D, Shaffer T (2009). International Conference on Medical Image Computing and Computer-Assisted Intervention.

[10] Liao Y, Yin G, Wang X, Zhong P, Fan X, Huang C (2019). Identification of candidate genes associated with the pathogenesis of small cell lung cancer via integrated bioinformatics analysis. Oncol Lett.

[11] Zawadzka I, Jeleń A, Pietrzak J, Żebrowska-Nawrocka M, Michalska K, Szmajda-Krygier D, Mirowski M, Łochowski M, Kozak J, Balcerczak E (2020). The impact of ABCB1 gene polymorphism and its expression on non-small-cell lung cancer development, progression and therapy: Preliminary report. Sci Rep.

[12] Zhang F, Cheng D, Wang S, Zhu J (2016). Human specific regulation of the telomerase reverse transcriptase gene. Genes (Basel).

[13] Dratwa M, Wysoczańska B, Łacina P, Kubik T, Bogunia-Kubik K (2020). TERT: Regulation and roles in cancer formation. Front Immunol.

[14] Barthel F P, Wei W, Tang M, Martinez-Ledesma E, Hu X, Amin S B, Akdemir K C, Seth S, Song X, Wang Q, Lichtenberg T, Hu J, Zhang J, Zheng S, Verhaak R G W (2017). Systematic analysis of telomere length and somatic alterations in 31 cancer types. Nat Genet.

[15] Leão R, Apolónio J D, Lee D, Figueiredo A, Tabori U, Castelo-Branco P (2018). Mechanisms of human telomerase reverse transcriptase (hTERT) regulation: Clinical impacts in cancer. J Biomed Sci.

[16] Dogan F, Forsyth N R (2021). Telomerase regulation: A role for epigenetics. Cancers (Basel).

[17] Ferreira M S V, Sørensen M D, Pusch S, Beier D, Bouillon A, Kristensen B W, Brümmendorf T H, Beier C P, Beier F (2020). Alternative lengthening of telomeres is the major telomere maintenance mechanism in astrocytoma with isocitrate dehydrogenase 1 mutation. J Neurooncol.

[18] Heidenreich B, Kumar R (2017). TERT promoter mutations in telomere biology. Mutat Res Rev Mutat Res.

[19] Xu Y, Goldkorn A (2016). Telomere and telomerase therapeutics in cancer. Genes (Basel).

[20] Autexier C, Lue N F (2006). The structure and function of telomerase reverse transcriptase. Annu Rev Biochem.

[21] Nakamura T M, Morin G B, Chapman K B, Weinrich S L, Andrews W H, Lingner J, Harley C B, Cech T R (1997). Telomerase catalytic subunit homologs from fission yeast and human. Science.

[22] Colebatch A J, Dobrovic A, Cooper W A (2019). TERT gene: Its function and dysregulation in cancer. J Clin Pathol.

[23] Campa D, Rizzato C, Stolzenberg-Solomon R, Pacetti P, Vodicka P, Cleary S P, Capurso G, Bueno-de-Mesquita H B As, Werner J, Gazouli M, Butterbach K, Ivanauskas A, Giese N, Petersen G M (2015). TERT gene harbors multiple variants associated with pancreatic cancer susceptibility. Int J Cancer.

[24] Stoehr R, Taubert H, Zinnall U, Giedl J, Gaisa N T, Burger M, et al (2015). Frequency of TERT promoter mutations in prostate cancer. Pathobiology.

[25] Bayram S, Ülger Y, Sümbül A T, Kaya B Y, Genç A, Rencüzoğullari E, Dadaş E (2016). Polymorphisms in human telomerase reverse transcriptase (h TERT ) gene and susceptibility to gastric cancer in a Turkish population: Hospital-based case-control study. Gene.

[26] Liu R, Xing M (2016). TERT promoter mutations in thyroid cancer. Endocr Relat Cancer.

[27] Wu Y, Yan M, Li J, Li J, Chen Z, Chen P, Li B, Chen F, Jin T, Chen C (2017). Genetic polymorphisms in TERT are associated with increased risk of esophageal cancer. Oncotarget.

[28] Wan S, Liu X, Hua W, Xi M, Zhou Y, Wan Y (2021). The role of telomerase reverse transcriptase (TERT) promoter mutations in prognosis in bladder cancer. Bioengineered.

[29] Llorca-Cardeñosa M J, Peña-Chilet M, Mayor M, Gomez-Fernandez C, Casado B, Martin-Gonzalez M, Carretero G, Lluch A, Martinez-Cadenas C, Ibarrola-Villava M, Ribas G (2014). Long telomere length and a TERT-CLPTM1 locus polymorphism association with melanoma risk. Eur J Cancer.

[30] Zhu C Q, Cutz J C, Liu N, Lau D, Shepherd F A, Squire J A, Tsao M - S (2006). Amplification of telomerase (hTERT) gene is a poor prognostic marker in non-small-cell lung cancer. Br J Cancer.

[31] Liu Z, Ma H, Wei S, Li G, Sturgis E M, Wei Q (2011). Telomere length and TERT functional polymorphisms are not associated with risk of squamous cell carcinoma of the head and necktelomere length, TERT genetic variations, and head and neck cancer risk. Cancer Epidemiol Biomarkers Prev.

[32] Wu H, Qiao N, Wang Y, Jiang M, Wang S, Wang C, Hu L (2013). Association between the telomerase reverse transcriptase (TERT) rs2736098 polymorphism and cancer risk: Evidence from a case-control study of non-small-cell lung cancer and a meta-analysis. PLoS One.

[33] Yoon K - A, Park J H, Han J, Park S H, Lee G K, Han J -Y, Zo J I, Kim J, Lee J E, Takahashi A, Kubo M, Nakamura Y, Lee J S (2010). A genome-wide association study reveals susceptibility variants for non-small cell lung cancer in the Korean population. Hum Mol Genet.

[34] Xing Y, Liu F, Li J, Lin J, Zhu G, Li M, Zhang C, Niu Y (2016). Case-control study on impact of the telomerase reverse transcriptase gene polymorphism and additional single nucleotide polymorphism (SNP)-SNP interaction on non-small cell lung cancers risk in Chinese Han population. J Clin Lab Anal.

[35] Lawi Z K, Al-Shuhaib M B S, Amara I B (2023). The rs1801280 SNP is associated with non-small cell lung carcinoma by exhibiting a highly deleterious effect on N-acetyltransferase 2. J Cancer Res Clin Oncol.

[36] Lawi Z K, Al-Shuhaib M B S, Amara I B, Alkhammas A H (2022). Two missense variants of the epidermal growth factor receptor gene are associated with non small cell lung carcinoma in the subjects from Iraq. Mol Biol Rep.

[37] Al-Shuhaib M B S (2018). A minimum requirements method to isolate large quantities of highly purified DNA from one drop of poultry blood. J Genet.

[38] Ye J, Coulouris G, Zaretskaya I, Cutcutache I, Rozen S, Madden T L (2012). Primer-BLAST: A tool to design target-specific primers for polymerase chain reaction. BMC Bioinformatics.

[39] Badi M A, Al-Shuhaib M B S, Aljubouri T R S, Al-Thuwaini T M, Dawud H H, Hussein T H, Al-Nafii A T, Abdulmalek D, Altamemi M K A, Fadhil I A, Albakri A H, Hashim H O (2021). Rapid and optimized protocol for efficient PCR-SSCP genotyping for wide ranges of species. Biologia (Bratisl).

[40] Ho H O, Al-Shuhaib M B S (2019). Exploring the potential and limitations of PCR-RFLP and PCR-SSCP for SNP detection: A review. J Appl Biotechnol Reports.

[41] Byun S O, Fang Q, Zhou H, Hickford J G H (2009). An effective method for silver-staining DNA in large numbers of polyacrylamide gels. Anal Biochem.

[42] Schoonjans F, Zalata A, Depuydt C E, Comhaire F H (1995). MedCalc: A new computer program for medical statistics. Comput Methods Programs Biomed.

[43] Barrett J C, Fry B, Maller J, Daly M J (2005). Haploview: Analysis and visualization of LD and haplotype maps. Bioinformatics.

[44] Barta J A, Powell C A, Wisnivesky J P (2019). Global epidemiology of lung cancer. Ann Glob Health.

[45] Alsubaie L M, Alsuwat H S, Almandil N B, Alsulaiman A, Abdulazeez S, Borgio F J (2020). Risk Y-haplotypes and pathogenic variants of Arab-ancestry boys with autism by an exome-wide association study. Mol Biol Rep.

[46] Ye G, Tan N, Meng C, Li J, Jing L, Yan M, Jin T, Chen F (2017). Genetic variations in TERC and TERT genes are associated with lung cancer risk in a Chinese Han population. Oncotarget.

[47] Gao L, Thakur A, Liang Y, Zhang S, Wang T, Chen T, Meng J, Wang L, Wu F, Jin T, Li X, Liu J J, Chen C, Chen M (2014). Polymorphisms in the TERT gene are associated with lung cancer risk in the Chinese Han population. Eur J Cancer Prev.

[48] Lan Q, Cawthon R, Gao Y, Hu W, Hosgood H, Barone-Adesi F, Ji B, Bassig B, Chow W, Shu X, Cai Q, Xiang Y, Berndt S, Kim C, Chanock S, Zheng W, Rothman N (2013). Longer telomere length in peripheral white blood cells is associated with risk of lung cancer and the rs2736100 (CLPTM1L-TERT) polymorphism in a prospective cohort study among women in China. PLoS One.

[49] Wang H - M, Zhang X - Y, Jin B (2013). TERT genetic polymorphism rs2736100 was associated with lung cancer: A meta-analysis based on 14,492 subjects. Genet Test Mol Biomarkers.

[50] Nie W, Zang Y, Chen J, Xiu Q (2014). TERT rs2736100 polymorphism contributes to lung cancer risk: A meta-analysis including 49,869 cases and 73,464 controls. Tumour Biol.

[51] Bae E Y, Lee S Y, Kang B K, Lee E J, Choi Y Y, Kang H (2012). Replication of results of genome wide association studies on lung cancer susceptibility loci in a Korean population. Respirology.

[52] Hosgood H, Menashe I, Shen M, Yeager M, Yuenger J, Rajaraman P, He X, Chatterjee N, Caporaso N E, Zhu Y, Chanock S J, Zheng T, Lan Q (2008). Pathway-based evaluation of 380 candidate genes and lung cancer susceptibility suggests the importance of the cell cycle pathway. Carcinogenesis.

[53] Choi J E, Kang H - G, Jang J S, Choi Y Y, Kim M J, Kim J S (2009). Polymorphisms in telomere maintenance genes and risk of lung cancer telomere maintenance gene polymorphisms in lung cancer. Cancer Epidemiol Biomarkers Prev.

[54] Rafnar T, Sulem P, Stacey S N, Geller F, Gudmundsson J, Sigurdsson A (2009). Sequence variants at the TERT-CLPTM1L locus associate with many cancer types. Nat Genet.

[55] Walsh K M, Gorlov I P, Hansen H M, Wu X, Spitz M R, Zhang H, Lu E Y, Wenzlaff A S, Sison J D, Wei C, Lloyd S M, Chen W, Frazier M L, Seldin M F, Bierut L J (2013). Fine-mapping of the 5p15.33, 6p22.1-p21.31, and 15q25.1 regions identifies functional and histology-specific lung cancer susceptibility loci in African-Americans. Cancer Epidemiol Biomarkers Prev.

[56] van Dyke A L, Cote M L, Wenzlaff A S, Abrams J, Land S, Iyer P, Schwartz A G (2009). Chromosome 5p region SNPs are associated with risk of NSCLC among women. J Cancer Epidemiol.

[57] Morais M, Dias F, Resende T, Nogueira I, Oliveira J, Maurício J, Teixeira A L, Medeiros R (2020). Leukocyte telomere length and hTERT genetic polymorphism rs2735940 influence the renal cell carcinoma clinical outcome. Future Oncol.

[58] Zhou M, Jiang B, Xiong M, Zhu X (2018). Association between TERT rs2736098 polymorphisms and cancer risk: A meta-analysis. Front Physiol.

[59] Fachiroh J, Sangrajrang S, Johansson M, Renard H, Gaborieau V, Chabrier A, Chindavijak S, Brennan P, McKay J D (2012). Tobacco consumption and genetic susceptibility to nasopharyngeal carcinoma (NPC) in Thailand. Cancer Causes Control.

[60] Yuan X, Cheng G, Yu J, Zheng S, Sun C, Sun Q, Li K, Lin Z, Liu T, Li P, Xu Y, Kong F, Bjorkholm M, Xu D (2017). The TERT promoter mutation incidence is modified by germline TERT rs2736098 and rs2736100 polymorphisms in hepatocellular carcinoma. Oncotarget.

[61] Wang S, Wu J, Hu L, Ding C, Kan Y, Shen Y, Chen X, Shen H, Guo X, Hu Z (2012). Common genetic variants in TERT contribute to risk of cervical cancer in a Chinese population. Mol Carcinog.

[62] Hashemi M, Amininia S, Ebrahimi M, Hashemi S M, Taheri M, Ghavami S (2014). Association between hTERT polymorphisms and the risk of breast cancer in a sample of Southeast Iranian population. BMC Res Notes.

[63] Landrum M J, Lee J M, Riley G R, Jang W, Rubinstein W S, Church D M, Maglott D R (2014). ClinVar: Public archive of relationships among sequence variation and human phenotype. Nucleic Acids Res.

[64] Logette E, Wotawa A, Solier S, Desoche L, Solary E, Corcos L (2003). The human caspase-2 gene: Alternative promoters, pre-mRNA splicing and AUG usage direct isoform-specific expression. Oncogene.

[65] Druillennec S, Dorard C, Eychène A (2012). Alternative splicing in oncogenic kinases: From physiological functions to cancer. J Nucleic Acids.

[66] Kirchner S, Cai Z, Rauscher R, Kastelic N, Anding M, Czech A, et al. (2017). Alteration of protein function by a silent polymorphism linked to tRNA abundance. PLoS Biol.

[67] Sauna Z E, Kimchi-Sarfaty C, Ambudkar S V, Gottesman M M (2007). Silent polymorphisms speak: How they affect pharmacogenomics and the treatment of cancer. Cancer Res.

[68] Martin K C, Ephrussi A (2009). MRNA localization: Gene expression in the spatial dimension. Cell.

